# Heterozygous *Ambra1* Deficiency in Mice: A Genetic Trait with Autism-Like Behavior Restricted to the Female Gender

**DOI:** 10.3389/fnbeh.2014.00181

**Published:** 2014-05-16

**Authors:** Ekrem Dere, Liane Dahm, Derek Lu, Kurt Hammerschmidt, Anes Ju, Martesa Tantra, Anne Kästner, Kamal Chowdhury, Hannelore Ehrenreich

**Affiliations:** ^1^Clinical Neuroscience, Max Planck Institute of Experimental Medicine, Göttingen, Germany; ^2^DFG Center for Nanoscale Microscopy and Molecular Physiology of the Brain (CNMPB), Göttingen, Germany; ^3^Cognitive Ethology Laboratory, German Primate Center, Göttingen, Germany; ^4^Department of Molecular Cell Biology, Max Planck Institute of Biophysical Chemistry, Göttingen, Germany

**Keywords:** Ambra1, autophagy, heterozygous null mutant mice, autism composite score, social interaction, ultrasound communication, repetitive behavior, cognitive rigidity

## Abstract

Autism-spectrum disorders (ASD) are heterogeneous, highly heritable neurodevelopmental conditions affecting around 0.5% of the population across cultures, with a male/female ratio of approximately 4:1. Phenotypically, ASD are characterized by social interaction and communication deficits, restricted interests, repetitive behaviors, and reduced cognitive flexibility. Identified causes converge at the level of the synapse, ranging from mutation of synaptic genes to quantitative alterations in synaptic protein expression, e.g., through compromised transcriptional or translational control. We wondered whether reduced turnover and degradation of synapses, due to deregulated autophagy, would lead to similar phenotypical consequences. Ambra1, strongly expressed in cortex, hippocampus, and striatum, is a positive regulator of Beclin1, a principal player in autophagosome formation. While homozygosity of the *Ambra1* null mutation causes embryonic lethality, heterozygous mice with reduced Ambra1 expression are viable, reproduce normally, and lack any immediately obvious phenotype. Surprisingly, comprehensive behavioral characterization of these mice revealed an autism-like phenotype in *Ambra1*^+/−^ females only, including compromised communication and social interactions, a tendency of enhanced stereotypies/repetitive behaviors, and impaired cognitive flexibility. Reduced ultrasound communication was found in adults as well as pups, which achieved otherwise normal neurodevelopmental milestones. These features were all absent in male *Ambra1*^+/−^ mice. As a first hint explaining this gender difference, we found a much stronger reduction of Ambra1 protein in the cortex of *Ambra1*^+/−^ females compared to males. To conclude, *Ambra1* deficiency can induce an autism-like phenotype. The restriction to the female gender of autism-generation by a defined genetic trait is unique thus far and warrants further investigation.

## Introduction

Autism-spectrum disorders (ASD) are heterogeneous neurodevelopmental disorders of yet poorly understood etiology that are characterized by severe socio-communicative deficits, restricted interests, stereotypies, and repetitive behaviors (Kanner, 1943; Asperger, 1944; American Psychiatric Association, 2013). Males are affected 4.3 times more frequent as compared to females (Kirkovski et al., 2013). Over the past decade, evidence has accumulated indicating that synaptic dysfunction, including changes in neurotransmission, plays a crucial role in the pathophysiology of ASD. In fact, mutations of genes encoding for synaptic proteins including neuroligins, neurexins, CNTNAP2, and SHANK3 have been associated with monogenic heritable ASD (Kumar and Christian, 2009; Huguet et al., 2013). Knockout of the eukaryotic translation initiation factor 4E-binding protein 2, an eIF4E repressor, or eIF4E overexpression leads to an increase in neuroligins and ASD-like phenotypes (Gkogkas et al., 2013). Essentially, the etiology of ASD directly or indirectly converges at the synapse, introducing the term “synaptopathy” for ASD (Zoghbi and Bear, 2012; Delorme et al., 2013).

Since causes of synaptic protein disbalance can be multifactorial, we wondered whether deregulated autophagy would also lead to ASD-like features. Autophagy is a self-degradative process controlling basal turnover of cellular components including synapses. It is involved in both pro-survival and pro-death mechanisms in different physiological and pathological conditions (Wirawan et al., 2012).

*Ambra1* (activating molecule in Beclin1-regulated autophagy) is a positive regulator of a principal mediator of autophagosome formation, Beclin1. In the postnatal mouse brain, strong Ambra1 expression is found in cortex, hippocampus, and striatum (Fimia et al., 2007). Even though Ambra1 has been visualized in the cytoplasm of pyramidal neurons of the hippocampal formation (Sepe et al., 2014), the exact cellular and subcellular localization of Ambra1 remains to be determined. In line with its important function in neurodevelopment, *Ambra1* null mutation causes autophagy impairment, neural tube defects, accumulation of ubiquitinated proteins, unbalanced cell proliferation, excessive apoptotic cell death, and ultimately embryonic lethality (Fimia et al., 2007).

Interestingly, a recent genome-wide association study on schizophrenia identified a genetic risk variation in a region on chromosome 11 (11p11.2) containing *AMBRA1* (Rietschel et al., 2012). In this region, five single nucleotide polymorphisms were associated with schizophrenia at odd ratios ranging from 1.24 to 1.25.

Autism-spectrum disorders and schizophrenia show a considerable syndromic overlap, including deficits in social cognition and communication, and like ASD, at least a subgroup of schizophrenia is regarded as a disease of the synapse (Eack et al., 2013). However, any potential role of *Ambra1* mutations in the expression of a complex behavioral phenotype reminiscent of schizophrenia or ASD as classical neurodevelopmental disorders has not been explored yet.

The present paper has therefore been designed to comprehensively characterize at the behavioral level mice with a heterozygous mutation of *Ambra1*, leading to a truncated, non-functional Ambra1 protein (via insertion of a gene-trapping vector into the mouse *Ambra1* gene) (Fimia et al., 2007). Indeed, we show here that (1) *Ambra1*^+/−^ mice display autism-like symptoms, including social interaction and communication deficits, repetitive behaviors, and cognitive rigidity. (2) Surprisingly, this autism-like phenotype is strongly observable only in female but not in male mice, and becomes evident before puberty. (3) Analysis of cortical brain tissue revealed a more prominent relative reduction of Ambra1 protein from WT level in *Ambra1*^+/−^ females as compared to males. Thus, *Ambra1*^+/−^ partial loss-of-function mutation is the first monogenic animal model of a predominantly female ASD.

## Materials and Methods

### Generation, genotyping, and housing of *Ambra1*^+/−^ and WT mice

All experiments were approved by the local animal care and use committee in accordance with the German animal protection law. The generation of functional *Ambra1* null mutant mice via inactivation of the *Ambra1* gene has been described in detail elsewhere (Fimia et al., 2007). *Ambra1*^+/−^ and wildtype (WT, *Ambra1*^+^*^/^*^+^) littermates of both genders with a >99% C57BL/6N genetic background were used for behavioral and biochemical studies. They were obtained from male *Ambra1*^+/−^ × female WT C57BL/6N breeding pairs. Genotypes of the offspring were analyzed by PCR of tail genomic DNA using the following primers: *Ambra1* forward primer 5′-GAA AAG CTC CCC ATC TTT TCT T-3′, *Ambra1* reverse primer 5′-ATC CCA AGG GCA GTA GAG TTC-3′ (yielding a 3 kb product in Ambra1^+/−^), interleukin-2 (*IL-2*) forward primer 5′-CTA GGC CAC AGA ATT GAA AGA TCT-3′, and *IL-2* reverse primer 5′-GTA GGT GGA AAT TCT AGC ATC ATC C-3′ (yielding 350 bp product in all samples for an internal control). PCR amplification of the DNA was carried out with Failsafe enzyme with PreMix D (Epicentre, Madison, WI, USA) under the following conditions: 3 min, 94°C (1 cycle); 30 s, 94°C; 45 s, 57°C; 2 min 30 s, 72°C (40 cycles), followed by final extension at 72°C for 5 min. Final PCR products were run on a 1.5% agarose gel and stained with ethidium bromide.

### Behavioral characterization of mice

For behavioral testing, mice were housed in groups of 3–5 (except where otherwise specified) in standard plastic cages, with food and water *ad libitum*. The temperature in the colony room was maintained at 20–22°C, with a 12 h light–dark cycle (lights on at 7:00 a.m.). All behavioral experiments were conducted by investigators, unaware of the genotype (“blinded”), during the light phase of the day (between 8:00 a.m. and 5:00 p.m.). Basic behavioral functions were assessed in two large consecutive cohorts of male and female mice (genders tested separately), always using littermate controls in a most balanced fashion. Tests were performed in the following order: elevated plus-maze, open field, hole board, rotarod, pre-pulse inhibition (PPI) of the startle response, hearing (startle curve upon random presentation of stimulus intensities from 65 to 120 dB), visual cliff, marble burying, Y-maze, a modified version of social interaction in the tripartite chamber (Moy et al., 2004), olfaction (buried food finding), sucrose preference, social interaction in pairs, Morris water-maze, hot plate, LABORAS home cage activity, novel object recognition, forced swim test, nest building, and vocalization. The age of mice at the beginning of testing was 11–12 weeks. Inter-test interval varied depending on the degree of “test invasiveness” but was at least 1 day. Comprehensive description of all tests used has been provided earlier (Radyushkin et al., 2010; Bodda et al., 2013; El-Kordi et al., 2013). In the following, only the most relevant tests for the autism-like behavioral phenotype are described in detail. After completion of the behavioral test battery, the mice were sacrificed at the age of 59–60 weeks. The cortex was dissected out and for biochemical analyses stored at −80°C.

### Sensory functions

#### Visual cliff test – vision

Visual integrity was tested in an apparatus consisting of a Perspex box (70 cm × 35 cm × 30 cm) with a transparent floor. The box was placed on the edge of a laboratory bench, in a way that 50% of the base was positioned on the bench (“ground” side), while the other half was extended over the edge of the bench, suspended 1 m above the floor (“air” side). Mice were then individually placed into the center of the base. The animal’s behavior was registered over a period of 5 min using a video-tracking system (Viewer2, Biobserve, Germany). The percentage of time spent on the “ground” versus “air” side of the box was calculated for each mouse. The animals were tested at the age of 11–12 weeks.

#### Buried food finding test – olfaction

The animals were first habituated to the transparent testing cages (29.5 cm × 18.5 cm × 13 cm) for 3 days with two trials of 20 min duration per day. Starting on day 3 of habituation, mice were deprived of food for 24 h before testing and received a piece of chocolate cookie (1.6 g) during each habituation trial and three to five cookies in their home cage after testing. After the daily habituation trials in the test cages, the animals received access to food in their home cages for 1 h. This procedure was continued until day 6. The test trials were performed on day 7. The mice had to locate a piece of chocolate cookie that was hidden approximately 1.5 cm below fresh bedding close to the wall at one end of the cage. The mouse was placed into the right corner at the opposite end of the cage, and the latency to locate the cookie and to start burying for it was measured with a cut-off time of 3 min. The animal was removed from the test cage after the cookie had been discovered and before it was consumed. In order to test whether *Ambra1* deficiency had altered the motivation to approach and consume the cookie, a visible test trial was performed after the hidden test. Here, the cookie was placed on top of the bedding. Generally, the latency to locate the cookie is significantly lower under the visible as compared to the hidden condition. Fresh cages and bedding were used for every habituation and test trials. Animals were tested at the age of 19–20 weeks.

#### Acoustic startle response – hearing

In this test, the startle reaction to an acoustic stimulus (pulse) that induces a movement of a force-sensitive platform was recorded over a period of 100 ms, beginning with the onset of the pulse. An experimental session consisted of a 2 min habituation period to a 65 dB background white noise (continuous throughout the session), followed by a baseline recording for 1 min at this background noise. After baseline recording, stimuli of different intensity and a fixed duration of 40 ms were presented. Stimulus intensity was varied between 65 and 120 dB, such that 19 intensities (in steps of 3 dB) from this range were used. Each stimulus intensity was presented 10× in a pseudorandom order with an inter-stimulus interval of 8–22 s. The amplitude of the startle response (expressed in arbitrary units) was defined as the difference between the maximum force detected during a recording window and the force measured immediately before stimulus onset. For each mouse, the amplitudes of the startle responses were averaged for every stimulus intensity. Animals were tested at the age of 11–12 weeks.

#### Hot plate test – pain perception

The hot plate test was used as a measure of pain perception. Mice were placed on a metal plate (Ugo Basile Srl, Comerio, Italy) that was preheated up to 55°C. The latency to show hind paw licking or jumping was recorded. Immediately after showing the response, the mice were removed from the platform. A cut-off time of 40 s ensured that the mice were not injured. Animals were tested at the age of 27–28 weeks.

### Spontaneous and novelty-induced activity

#### LABORAS – spontaneous home cage behavior

The LABORAS system consists of a triangular shaped sensor platform (Carbon Fiber Plate 1000 mm × 700 mm × 700 mm × 30 mm, Metris B.V., Hoofddorp, Netherlands), positioned on two orthogonally placed force transducers (Single Point Load Cells) and a third fixed point attached to a heavy bottom plate (Corian Plate 980 mm × 695 mm × 695 mm × 48 mm). The whole structure stands on three spikes, which are adjustable in height and can absorb external vibrations. Mice are housed in clear polycarbonate cages (Makrolon type II cage, 22 cm × 16 cm × 14 cm) with a wood-chip bedding covered floor. The cage is placed directly onto the sensing platform, with the upper part of the cage (including the top, food hopper, and drinking bottle) suspended in a height-adjustable frame separate from the sensing platform. Resulting electrical signals caused by the mechanical vibrations of the movement of the animal are transformed by each force transducer, amplified to a fixed signal range, filtered to eliminate noise, digitized, and stored on a computer. The computer then processes the stored data using several signal analysis techniques to classify the signals into the behavioral categories of eating, drinking, scratching, circling, climbing, immobility, locomotion, and grooming. The behavior that dominates is scored. Spontaneous mouse behavior was assessed from 5:00 p.m. until 9:00 a.m., with 1 h habituation to the cages before the initiation of recording. Animals were tested at the age of 27–28 weeks.

#### Open field – exploratory activity

Exploratory activity in a novel environment was tested in a gray circular Perspex arena (120 cm in diameter, surrounded by a wall of 25 cm height). Individual animals were placed into the center of the open field and allowed to explore it for 7 min. The exploratory behavior of the mouse was recorded using a tracking-software (Viewer2, Biobserve, Germany). The distance traveled (millimeter) and the time spent (second) in the central, intermediate, and peripheral zones of the open field was analyzed. Animals were tested at the age of 11–12 weeks.

#### Hole board – exploratory activity

Individual mice were placed into the center of the hole board (transparent Perspex chamber (50 cm × 50 cm × 36 cm), with a non-transparent floor raised 3 cm above the bottom of the chamber with 16 equally spaced holes of 2.2 cm diameter), and allowed to explore the chamber for 5 min. The number of holes explored (head dips) was monitored by two layers of infrared photo beams connected to a computer with the AKS software (TSE Systems, Bad Homburg, Germany). Animals were tested at the age of 11–12 weeks.

### Motor coordination, motor learning, and sensorimotor gating

#### Rotarod – motor coordination and learning

The rotarod (Ugo Basile Srl, Comerio, Italy) consists of a horizontal rotating drum that was accelerated from 4 to 40 rpm over the course of 5 min. Individual mice were placed on the drum, and once they were balanced, the drum was accelerated. The time (second) at which the animal fell from the drum was recorded using a trip switch. Each animal was tested on two consecutive days. Animals were tested at the age of 11–12 weeks.

#### Pre-pulse inhibition of the startle response – sensorimotor gating

Animals were placed in small metal cages (82 mm × 40 mm × 40 mm) to restrict major movements and exploratory behavior. The cages were equipped with a movable platform floor attached to a sensor that recorded vertical movements of the floor. The cages were placed in four sound attenuating cabinets (TSE Systems, Bad Homburg, Germany). Startle reflexes were evoked by acoustic stimuli delivered by a loudspeaker that was suspended above the cage and connected to an acoustic generator. The startle reaction to an acoustic stimulus that induces a movement of a force-sensitive platform was recorded over a period of 260 ms beginning with the onset of the pulse. An experimental session consisted of a 2-min habituation period to a 65 dB background white noise (continuous throughout the session), followed by a baseline recording for 1 min at background noise. After baseline recording, six pulse-alone trials using startle stimuli of 120 dB intensity and 40 ms duration were applied to decrease the influence of within-session habituation. These data were not included in the 120 dB/40 ms analysis of the PPI. For tests of PPI, the startle pulse was applied alone or after a pre-pulse stimulus of 70, 75, or 80 dB intensity and 20 ms duration. A delay of 100 ms with background noise was interposed between the presentation of the pre-pulse and pulse stimulus. The trials were presented in a pseudorandom order with a variable interval ranging from 8 to 22 s. The amplitude of the startle response (expressed in arbitrary units) was defined as the difference between the maximum force detected during the recording window and the force measured immediately before the stimulus onset. For each animal, the amplitudes were averaged separately for the two types of trials (i.e., stimulus alone or stimulus preceded by a pre-pulse). PPI was calculated as the percentage of the startle response using the following formula: % PPI = 100−[(startle amplitude after pre-pulse)/(startle amplitude after pulse only) × 100]. Animals were tested at the age of 12–13 weeks.

### Emotionality

#### Elevated plus-maze – anxiety-like behavior

Individual animals were placed on the central platform facing an open arm of the plus-maze (made of gray Perspex with a 5 cm × 5 cm central platform, two open and two walled arms with the following dimensions: 30 cm × 5 cm × 15 cm; illumination density 135 lx). The animal’s behavior was recorded for 5 min by an overhead video camera and a computer equipped with automated tracking-software (Viewer2, Biobserve, Germany) to calculate the time the animal spent in open or walled arms. The time spent on walled versus open arms was used as an index of anxiety- versus anxiolytic-like behavior. Animals were tested at the age of 11–12 weeks.

#### Sucrose preference test – anhedonia

The sucrose preference test was performed using a two-bottle choice setting, during which mice had free access to both water and a sucrose solution. Animals were first habituated for 48 h to consume water from the two small (100 ml) bottles. After habituation, mice were deprived of water, and the sucrose preference was measured over three consecutive days. The first 2 days served as a habituation to sucrose solution. The results of d3 were used for the evaluation of sucrose preference. Each day, group-housed mice were placed individually into small plastic cages and two bottles were presented to them for 60 min – one filled with tap water and another one containing a 2% sucrose solution. The amount of consumption of water or sucrose solution was determined by weighing the bottles before and after the session. The position of the bottle containing the sucrose solution was counterbalanced across the left and the right side of the cage, and for individual animals its position was alternated between tests. Sucrose preference (%) was calculated as follows: preference = 1/4[sucrose solution intake (ml)/total fluid intake (ml)] × 100. Animals were tested at the age of 23–24 weeks.

#### Forced swim test – depression-like behavior

The forced swim test was performed as initially described by (Porsolt et al., 1977). The mouse was placed for 6 min into a glass cylinder (height: 25 cm, diameter: 10 cm) filled with water (23–25°C) up to a height of 10 cm. A mouse was judged to be immobile when it floated in an upright position and made only minor movements to keep its head above the water level. After a habituation period of 2 min, the time spent immobile was measured during the last 4 min of the 6 min testing period. Animals were tested at the age of 39–40 weeks.

### Cognitive phenotyping

#### Novel object recognition – object memory

This test consists of a sample trial that is followed by a test trial. The mice were first habituated for 10 min to an open field (40 cm × 40 cm × 40 cm) made of gray plastic. During the sample trial, a novel object was introduced to the open field. There were two possible locations where the object could be positioned. The position of the novel object during the sample trial was counterbalanced over the animals tested. The mouse was allowed for 10 min to explore the novel object. Immediately after the sample trial, the mouse was subjected to a test trial. Here, the mouse was presented with the familiar object in its original position already known from the sample trial and a novel object and was allowed to freely explore the two objects for 10 min. Exploration of an object was assumed when the mouse approached an object and had physical contact with it, either with its vibrissae, snout, or forepaws. Vicinity to an object, at a distance <2 cm, was not considered as exploratory behavior. For each retention delay, the proportion of time exploring the novel object, relative to the total time spent exploring both objects, was taken as a measure of object recognition: recognition index = time novel/(time novel + time familiar). Novel object recognition index values higher than 0.5 suggest a preference for the novel object, values close to 0.5 would suggest no recognition, while values well below 0.5 suggest a preference for the familiar object. Animals were tested at the age of 29–30 weeks.

#### Y-maze continuous alternation – working memory

Spontaneous alternation performance was assessed in a Y-maze. The apparatus had an open roof and was constructed of gray Plexiglas with three arms (7.5 cm × 18 cm × 23.5 cm) radiating from a triangle-shaped central platform. Each animal received one trial in the Y-maze. Each trial lasted 5 min and began by placing the animal on the central platform, allowing it to freely explore the three arms. The entire apparatus was cleaned with 75% ethanol solution after each trial. An arm entry was scored when the mouse entered an arm with all four paws. The following parameters were calculated: (i) total number of entries, (ii) number of triplets: the number of consecutive choices of each of the three arms, without re-entries during the last three choices, and irrespective of the order of the chosen arms, and (iii) alternation-ratio: number of triplets divided by total number of entries minus 2. Animals were tested at the age of 15–16 weeks.

#### Morris water-maze – spatial learning and memory

A circular tank (diameter 1.2 m, depth 0.4 m) was filled with opaque water (25 ± 1°C, depth 0.3 m) and the escape platform (10 cm × 10 cm) was submerged 1 cm below the water level. The animals swim patterns were registered with a video-tracking system (Viewer2, Biobserve GmbH, Germany). Escape latency, swim speed, and path length were recorded for each trial.

Hidden platform acquisition: for 8 days, mice were trained to find a hidden platform which was submerged 0.5 cm below water level and positioned in the center of one of the four quadrants of the pool. For each mouse the location of the platform was fixed throughout the days of acquisition. Mice had to locate the hidden platform by using extra-maze cues placed on the walls of the testing room. Each mouse was subjected to four trials with an inter-trial interval of 5 min. Mice were released into the water facing the pool wall at one of four start locations and allowed to search for the platform for a maximum of 90 s. A mouse that failed to find the platform within 90 s was guided to the platform and remained there for 20 s before being removed from the pool.

Spatial probe (hidden platform): the day after the completion of the hidden platform training, a probe trial was conducted in order to determine whether the mice had developed a spatial bias for the former platform quadrant. The platform was removed from the pool and mice were allowed to swim for 90 s. The percentage of time spent in each quadrant of the pool was recorded.

Reversal learning: in order to investigate cognitive flexibility, a reversal learning task was performed. The experimental procedure was identical to the one used for the hidden platform training with the exception that the escape platform was moved from the original position to a different quadrant. Reversal learning was tested for 4 days followed by a probe trial. Animals were tested at the age of 23–24 weeks.

### Autism-related behaviors

#### Communication – ultrasound vocalization

Ultrasonic vocalizations (USVs) of mice were recorded with a microphone, connected to a preamplifier, and analyzed at a sampling frequency of 300 kHz using the Avisoft Recorder 4.2 software (Avisoft Bioacoustics, Berlin, Germany). Prior to recordings, mice (males and females) were housed for 24 h in single cages. Recordings were made from a resident mouse (male or female) that was vocalizing in its home cage (3 min test duration) upon exposure to a female intruder mouse (Hammerschmidt et al., 2012). Male and female resident mice were tested with unfamiliar females anesthetized with an i.p. injection of 0.25% tribromoethanol in a volume of 0.1 ml/10 g. Number of calls per recording session was counted and USVs were separated from other sounds using the whistles detection algorithm of Avisoft-SASLab5.2 (Avisoft Bioacoustics, Berlin, Germany) with following selection criteria: possible changes per step = 4 (4687 Hz), minimal continuity = 8 ms, and possible frequency range = 35–150 kHz. These criteria had been tested in former studies of mouse USVs (Hammerschmidt et al., 2012; El-Kordi et al., 2013). Animals were tested at the age of 39–40 weeks.

#### Social competence – nest building

It is well known that nest construction is impaired in mouse models of ASD (Satoh et al., 2011; El-Kordi et al., 2013). Group-housed mice were transferred to single-housing 1 h before beginning of the dark phase. Cages for single-housing contained wood-chip bedding and nesting towels. After two nights of habituation, nesting towels were replaced by nestlets (pressed cotton squares weighing approximately 3 g). Nest building was assessed in the next morning by weighing the leftover material and using a rating scale ranging from 1 to 5 with lower scores indicating aberrant nest building behavior. Animals were tested at the age of 43–44 weeks.

#### Social approach – social interaction in pairs

The social interaction in pairs was performed in a neutral testing cage (gray Plexiglas box, 30 cm × 30 cm × 30 cm). Each mouse was habituated to the testing cage for 10 min on two consecutive days. One day after the last habituation trial, pairs of unfamiliar mice of the same genotype were placed into the testing cage for 10 min. The time the animals spent in close contact was recorded by a trained observer. Animals were tested at the age of 23–24 weeks.

#### Social interaction in the tripartite chamber

Sociability and social memory were tested in a rectangular box that was divided into three chambers (40 cm × 20 cm × 22 cm). The dividers were made from transparent Plexiglas and had rectangular entries (35 mm × 220 mm). The floor of the box was covered with wood-chip bedding that was exchanged between trials. The test mouse was introduced into the middle chamber, with the entries to the other two chambers closed, and allowed to acclimatize for 5 min. Thereafter, a small wire cage (140 mm × 75 mm × 60 mm) containing an unfamiliar male C57BL/6N mouse of the same age and weight (stranger 1) was placed in one outer chamber. An empty wire cage was positioned in the other outer chamber. The location (outer left or right chamber) of stranger 1 was alternated between trials. The stranger mice had been habituated to the wire cages for several days. After unblocking the entries to the outer chambers the test mouse was allowed to freely move between chambers for 10 min. Time spent in and number of entries into each chamber were recorded by a video-tracking system (Viewer2, Biobserve GmbH, Germany). Each mouse received a second and third trial. The second trial was identical to the first trial except that the stranger mouse was placed into the other outer chamber in order to control for a possible side bias. On the third trial, the test mouse was presented with the familiar stranger 1 and an unfamiliar stranger 2. A significant preference for the unfamiliar stranger 2 over the familiar stranger 1 indicates an intact social memory. A sociability and a social memory index were calculated as follows: sociability index = (time investigating stranger/time investigating stranger + time investigating empty cage) × 100; Memory index = (time investigating unfamiliar mouse/time investigating unfamiliar + familiar mouse) × 100. Animals were tested at the age of 19–20 weeks.

#### Stereotypic and compulsive behavior – marble burying test

This test is used to assess stereotypies and obsessive–compulsive behavior in mice. Mice were tested in plastic cages (34.5 cm × 56.5 cm × 18 cm) filled with 5 cm deep wood-chip bedding. Twenty-four glass marbles were placed on the surface of the bedding. The marbles were arranged in six rows with four marbles per row at a distance of 4 cm. Each mouse was placed into the cage (illumination 6 lx) and could freely manipulate the marbles for 30 min. The number of buried marbles (at least to 2/3 their depth) was scored. Animals were tested at the age of 15–16 weeks.

#### Stereotypic and repetitive behavior – circling

Frequency of circling was measured with the LABORAS homecage observation system as described in detail previously (Van de Weerd et al., 2001; El-Kordi et al., 2013).

### Neonatal testing

Ambra1^+/−^ male mice were mated with C57BL/6N female mice that had never given birth before (primipara). Approximately 2 weeks after pairing, females were individually housed and carefully inspected twice daily for pregnancy or delivery. The day of birth was noted as postnatal day (PND) 0. To identify individual pups, they were labeled using non-toxic tattoo ink on PND3. The ink was inserted subcutaneously through a 30 gage hypodermic needle tip into the center of the paw. As with adults, experiments were always performed by a trained observer, unaware of the genotype of the animals (“blinded”). Litters used for experiments contained 6–10 pups. For the assessment of developmental milestones and neurological reflexes, pups (male *Ambra1*^+^*^/^*^+^ *n* = 23, male *Ambra1*^+/−^ *n* = 22, female *Ambra1*^+^*^/^*^+^ *n* = 27, and female *Ambra1*^+/−^ *n* = 25) were tested daily between PND4 and PND21 (weaning at PND23). Each subject was tested at approximately the same time of day. The battery of tests performed provides an assessment of physical and neurodevelopmental milestones as well as neuromotor coordination throughout the neonatal period. The parameters measured are expressed and maturing at different periods throughout the first 21 days of life. Neonatal assessments comprise (i) maturation readouts describing physical development, (ii) neurodevelopmental measures based on neurological reflexes, and (iii) the achievement of neuromotor coordination (Vorhees et al., 1979; Heyser, 2004; Hill et al., 2008; Bodda et al., 2013).

#### Maturation readouts

Body weight development and the opening of eyes and ears are monitored daily.

#### Neurodevelopmental measures

##### Placing response

Pups are suspended in the air by grasping the pup gently around the trunk, making sure that none of the paws touched a solid surface. A thin metal bar is put in contact with the back of a paw. Starting from PND4, it is monitored with one trial per day whether the paws are raised (proper response). Criterion is reached when pups show the proper response on two consecutive days.

##### Surface righting reflex

Animals are placed on their back on a surface and then released. The time needed for each pup to right itself is recorded and the performance is monitored twice daily, starting from PND4. Criterion is reached when the pup can right itself within 2 s in both trials on two consecutive days.

##### Cliff avoidance

The pups are observed daily with one trial from PND6 until pup shows retraction (cliff avoidance reflex) within 10 s after being placed on an edge, with forepaws and nose just over the edge. Criterion is reached when pups show cliff avoidance reflex within 10 s on two consecutive days.

##### Negative geotaxis reflex

The pups are observed daily with one trial from PND7 by placing them on an inclined plane (30° angle) with head facing downwards. The time needed for the pup to change its orientation, so that its head faced up the incline (proper response) is measured. Response of each pup is observed for 30 s. Criterion is reached when the proper response appears before 30 s on two consecutive days.

##### Tactile startle

A puff of air (e.g., experimenter’s breath) is gently applied to the pups, starting on PND10. Criterion is reached when the proper response (jumping or running) is observed on three consecutive days.

##### Ear twitch

The cotton tip of an applicator is pulled out and the tip twisted to form a fine filament. The filament is gently brushed against the tip of the ear for three times. Criterion is reached when pups show the proper response of flattening the ear against the side of the head on three consecutive days.

##### Air righting reflex

The pup is held upside down with two fingers holding either side of the head and two fingers holding the hind quarters approximately 10 cm over a cage containing 5 cm of shavings. The pup is released and its position upon landing observed for one trial, starting from PND10 until criterion is reached, when the pup is able to turn around and land on the four paws over three consecutive days.

#### Neuromotor coordination measures

##### Open field traversal

The pup is placed in the center of a 13-cm circle and the time needed to escape off the circle is recorded, starting from PND10. The trial is terminated when after 30 s the pup remained in the circle. Criterion is reached when the time to move off the circle is <30 s on two consecutive days.

##### Wire suspension

Pups are forced to grasp a 3-mm wire and hang from it on their forepaws. Testing starts on PND10 onward until pups are able to hold the wire for 30 s. Criterion is reached when the pup is able to hang for 30 s on two consecutive days.

### Neonatal vocalization

Ultrasonic vocalizations of pups in response to the separation from their mothers were recorded on PND8-9 (isolation-induced vocalization). For recording, pups were selected randomly from their litter, weighed and placed in a sound-proof custom-made plastic box (diameter 13.5 cm). An ultrasound microphone (UltraSoundGate CM16) fixed in the lid of the box 12 cm above the bottom was connected to a preamplifier (UltraSoundGate 116) coupled to a notebook computer. Similar to the recordings made in adult mice (described above), USVs were separated from other sounds using the whistles detection algorithm of Avisoft-SASLab 5.2 with same selection criteria, except that the minimum continuity was set to 5 min for the neonatal recording. The total number of calls and calling duration were measured during an observation period of 3 min.

### Biochemical analyses

#### Real-time quantitative reverse transcription-PCR

Total RNA was isolated from cortex tissue of mice using miRNeasy Mini Kit (Qiagen, Hilden, Germany). The cDNA was synthesized from 1 μg of RNA using the SuperScript III Reverse Transcriptase (Life Technologies, Darmstadt, Germany), oligo-dT, and random N6 primers in a total volume of 20 μL. For quantitative reverse transcription-PCR (qPCR), 4 μl cDNA were used as template with 6 μl of Power SYBR Green PCR Master Mix (Life Technologies, Darmstadt, Germany) and 5 pmol of primers. The following primers were used: Ambra1 forward primer: 5′-AGG CTC CAG TGG TGG GAC TTC AC-3′, Ambra1 reverse primer: 5′-GCC AGG AGC TGA CCA TCT GCA G-3′, β-actin forward primer: 5′-CTT CCT CCC TGG AGA AGA GC-3′, β-actin reverse primer: 5′-ATG CCA CAG GAT TCC ATA CC-3′. qPCR reactions were run on LightCycler 480 System (Roche, Mannheim, Germany) with three technical replicates. Relative expression levels of Ambra1 were calculated using the threshold cycle method (LightCycler^®^ 480 Software release 1.5.0 SP3, Roche, Mannheim, Germany) and normalization to β-actin.

#### Protein extraction and Western blot

Total protein was extracted from cortex tissue. In brief, frozen tissue was homogenized in RIPA-lysis buffer (150 mM NaCl, 1.0% Triton X-100, 0.5% sodium deoxycholate, 0.1% SDS, 50 mM Tris, pH 7.4) with Halt Protease Inhibitor Single-Use Cocktail (Thermo Scientific, Waltham, MA, USA) using tissue ultra-mixer. The supernatant was collected after centrifugation at 12,000 rpm for 45 min at 4°C. The protein concentration was measured by Lowry assay. Protein lysates were denatured by boiling in a Laemmli buffer at 95°C for 5 min and stored at −80°C. For Western blot, 50 μg of protein was loaded on 8% SDS-PAGE and transferred onto nitrocellulose membrane (GE Healthcare, Buckinghamshire, UK) with 235 mA for 3 h. The membranes were blocked in 5% non-fat milk in Tris-buffered saline-Tween (TBST; 50 mM Tris, 150 mM NaCl, 0.5% Tween 20, pH 7.4) for 1 h at room temperature and incubated with primary antibodies diluted in 5% milk in TBST: anti-Ambra1 (1:1000; Merck Millipore, Darmstadt, Germany) and anti-actin (1:1000; Sigma-Aldrich, Taufkirchen, Germany) at 4°C overnight. After incubation with horseradish peroxidase conjugated secondary antibody diluted in 5% non-fat milk in TBST (1:5000; Sigma-Aldrich, Germany) for 1 h at room temperature, visualization was performed with an Immobilon Western Chemiluminescent HRP Substrate (Merck Millipore, Germany), followed by exposure to Amersham Hyperfilm ECL (GE Healthcare, UK). Densitometrical analysis of bands was performed using the public domain of the ImageJ program. Bands were measured by ImageJ. Ambra1 signals were all normalized to their respective actin signals and were expressed in % male WT. For the analysis of the Δ-value, male and female animals were expressed in %WT to the respective gender and 100% was subtracted (Δ-value = %WT−100%).

### Statistical procedures

All data were analyzed separately for males and females. Between-group comparisons were made by either one-way analysis of variance (ANOVA) with repeated measures or *t*-test for independent samples. Within-group tests of chance level performance using ratio or percentage calculations were performed via single-group *t*-tests against a chance level of either 0.25 or 0.5 when indicated. Mann–Whitney *U*, Wilcoxon, and Chi-square tests were used if the normality assumption was violated (as assessed by the Kolmogorov–Smirnov test) or in cases where between-group comparisons of *z*-transformed data were made. All statistics were performed using SPSS v.17 (San Diego, USA) or Prism Graph Pad software. The search and exclusion of significant outliers from single data sets (indicated from the visual inspection of the corresponding scatter plots) was performed using the Grubbs’ test. Data presented in the figures and text are expressed as mean ± SEM; *p*-values <0.05 were considered significant.

#### Autism composite score

For the autism composite score, selected single readouts measuring autism-like symptoms were *z*-standardized and presented such that higher values represent higher symptom severity. The *Z*-score transformation was performed to standardize single readouts to the same scale, generating variables with a mean of 0 and a standard deviation of 1. *Z*-standardization was performed for genders separately, but always included both genotypes, i.e., *Ambra1*^+/−^ and WT mice. The standard operating procedure (SOP) for the score calculation is given in Box 1. For calculating the composite score, relevant items are selected based on statistically significant (or close to significant) between-group comparisons. Social preference and social memory performance were included in the composite score as delta values or difference scores (time spent in mouse compartment−time spent in empty compartment; time spent with unfamiliar mouse−time spent with familiar mouse). A reliability analysis of the composite score was performed on the selected items by calculating Cronbach’s alpha as a measure of internal consistency. Gender-specific composite scores were obtained by integrating the means of the *z*-transformed behavioral readouts. Animals with missing values in the behavioral readouts selected for the composite score were excluded from data analysis. This exclusion concerned three males (+/+: *n* = 1; ±: *n* = 2) and four females (+/+: *n* = 3; ±: *n* = 1). Genotype-dependent group comparisons of composite scores and *z*-transformed readouts were conducted by Mann–Whitney *U*-tests. Intercorrelation patterns (pairwise Spearman correlations) were based on available sets of *z*-standardized raw scores. To see whether the autism composite score could be used to reliably predict the genotype of a given mouse (probability of a correct genotype assignment or diagnosis) a binary logistic regression analysis was performed separately for males and females.

Box 1 **Standard operating procedure for calculating the autism composite score with SPSS v.17.**

**Select Readouts**

Select autism-relevant readouts from your behavioral test battery. Readouts should cover all three general symptom categories, including communication, social functions, and stereotypic/repetitive behaviors.

**Recode Data**

If necessary, single readouts have to be recoded in a way that higher values correspond to higher levels of symptom severity, e.g., vocalization: number of calls. For example: animal 1: 10, animal 2: 45, animal 3: 97 → animal 1: 10 × −1, animal 2: 45 × −1, and animal 3: −97 × −1. Now animal 1 that exerted the lowest number of calls has the highest value of −10 and thus the highest degree of symptom severity (as compared to values of −45 or −97).

**Imputation of Missing Values**

If there are missing values, e.g., animal X has only valid values for tests 1, 2, 3, 4, 5, 6, 7, 9, and 10 but not 8, the missing value can be imputed in order to have a complete data set and one value for each readout and animal. However, missing values should not exceed 30% of the total number of values possible (e.g., animal X: 10 readouts and 3 missing values → imputation; animal Y: 10 readouts and 5 missing values → animal is excluded). In the SPSS program, the imputation procedure can be found under “Analyze” → “Analyze missing values” → “Multiple imputation” → “Impute missing values.” Enter “Imputation: 10” and then select the variables to be imputed and enter data-set name “Imputation.”

***Z*-Transformation**

The *z*-transformation procedure can be found under “Analyze” → “Descriptive statistics” → “Descriptives.” Then select the readouts/variables to be *z*-transformed and click “save standardized values as variables.”

**Composite Score**

To calculate the composite score go to “Transform” → “Compute variable.” Enter “Composite score” in the field “Target variable” and enter “mean (*X*_1_, *X*_2_, *X*_3_ … *X_n_*)” under “Numeric expression.” Here *X* refers to one of the *z*-transformed variables. Then click on OK and your composite score will appear in your SPSS-Matrix under the heading “Composite score.”

## Results

### Adult Ambra1^+/−^ mice show essentially normal basic behavior

**Figure 1:**
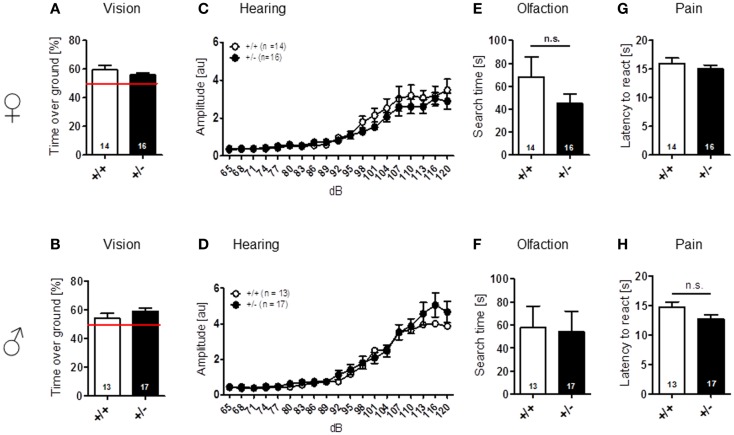
**Normal sensory function is found in male and female *Ambra1*^+/−^ versus *Ambra1*^+^*^/^*^+^ mice**. The upper row presents results for female, the lower row for male mice. **(A,B)** Visual cliff test; **(C,D)** hearing curve; **(E,F)** buried food finding; and **(G,H)** hot plate test. Mean ± SEM presented; respective sample sizes are indicated in the figures.

**Figure 2:**
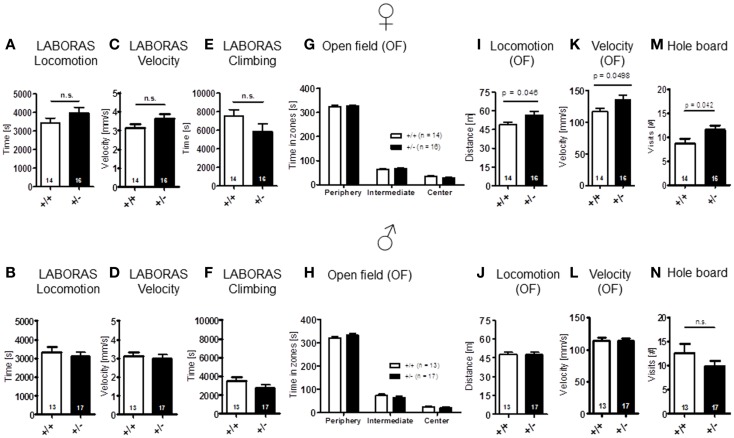
**Spontaneous and novelty-induced activity of *Ambra1*^+/−^ versus *Ambra1*^+^*^/^*^+^ mice are widely comparable**. The upper row presents results for female, the lower row for male mice. **(A–F)** LABORAS standard readouts; **(G–L)** open field; and **(M,N)** hole board. Female *Ambra1*^+/−^appear slightly more active. Mean ± SEM presented; respective sample sizes are indicated in the figures.

Sensory functions, i.e., vision [females: *t*(28) = 1.157, *p* = 0.257; males: *t*(28) = 1.131, *p* = 0.268], hearing [females: genotype: *F*(1, 28) = 0.689, *p* = 0.414, dB: *F*(18, 504) = 41.974, *p* < 0.001, genotype × dB: *F*(18, 504) = 0.717, *p* = 0.795; males: genotype: *F*(1, 28) = 0.562, *p* = 0.460, dB: *F*(18, 504) = 49.680, *p* < 0.001, genotype × dB: *F*(18, 504) = 0.578, *p* = 0.916], olfaction [females: *t*(28) = 1.261, *p* = 0.218; males: *t*(28) = 0.138, *p* = 0.891], and pain perception [females: *t*(28) = 0.771, *p* = 0.447; males: *t*(28) = 1.630, *p* = 0.114] were all found comparable in WT and *Ambra1*^+/−^ mice (Figure 1). Spontaneous and novelty-induced activity as observed in LABORAS, i.e., spontaneous home cage behavior [females: locomotion: *t*(28) = 1.351, *p* = 0.188, velocity: *t*(28) = 1.404, *p* = 0.171, climbing: *t*(28) = 1.589, *p* = 0.123; males: locomotion: *t*(28) = 0.573, *p* = 0.572, velocity: *t*(28) = 0.374, *p* = 0.711, climbing: *t*(28) = 1.369, *p* = 0.182] (Figures 2A–F), and time spent in different zones of the open field [females: time in zones: periphery: *t*(28) = 0.204, *p* = 0.840, intermediate: *t*(28) = 0.627, *p* = 0.535, center: *t*(28) = 1.798, *p* = 0.083; males: time in zones: periphery: *t*(28) = 1.071, *p* = 0.293, intermediate: *t*(28) = 1.053, *p* = 0.301, center: *t*(28) = 0.626, *p* = 0.537], were essentially comparable between genotypes (Figures 2G,H). Locomotion and running velocity in the open field (Figures 2I,K), as well as holes visited in hole board (Figure 2M) were slightly higher in female *Ambra1*^+/−^ [locomotion: *t*(29) = 2.084, *p* = 0.0461, velocity: *t*(28) = 2.050, *p* = 0.0498, hole board: *t*(28) = 2.127, *p* = 0.042] but not in male *Ambra1*^+/−^ mice (Figures 2J,L,N) [locomotion: *t*(28) = 0.051, *p* = 0.960, velocity: *t*(28) = 0.064, *p* = 0.950, hole board: *t*(28) = 1.317, *p* = 0.198], suggesting that general activity is slightly increased in female *Ambra1*^+/−^ mice.

**Figure 3:**
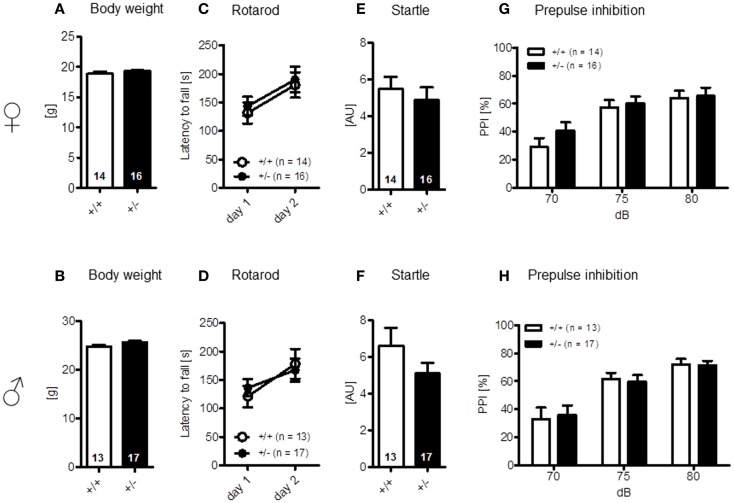
**Motor performance and sensorimotor gating of male and female *Ambra1*^+/−^ versus *Ambra1*^+^*^/^*^+^ mice are normal**. The upper row presents results for female, the lower row for male mice. **(A,B)** Body weight; **(C,D)** motor performance and motor learning; **(E,F)** startle response to a 120 db stimulus; and **(G,H)** pre-pulse inhibition. Mean ± SEM presented; respective sample sizes are indicated in the figures.

The LABORAS readout “circling” that has been proposed as a measure of stereotypic behavior in the mouse is presented separately under the section autism-specific behaviors (see below). Body weight [females: *t*(28) = 1.368, *p* = 0.182; males: *t*(28) = 1.744, *p* = 0.092] (Figures 3A,B) and motor coordination and balancing, i.e., performance on rotarod, including motor learning [females: genotype: *F*(1, 28) = 0.204, *p* = 0.655, day: *F*(1, 28) = 9.117, *p* = 0.005, genotype × day: *F*(1, 28) = 0.006, *p* = 0.940; males: genotype: *F*(1, 28) = 0.006, *p* = 0.937, day: *F*(1, 28) = 11.854, *p* = 0.002, genotype × day: *F*(1, 28) = 1.019, *p* = 0.321], as well as startle response [females: *t*(28) = 0.622, *p* = 0.539; males: *t*(28) = 1.140, *p* = 0.172] and sensorimotor gating (measured by PPI) were again similar in both genotypes [females: genotype: *F*(1, 28) = 0.529, *p* = 0.473, dB: *F*(2, 56) = 48.374, *p* < 0.001, genotype × dB: *F*(2, 56) = 1.420, *p* = 0.250; males: genotype: *F*(1, 28) = 0.0001, *p* = 0.991, dB: *F*(2, 56) = 45.917, *p* < 0.001, genotype × dB: *F*(2, 56) = 0.211, *p* = 0.810] (Figures 3C–H).

**Figure 4:**
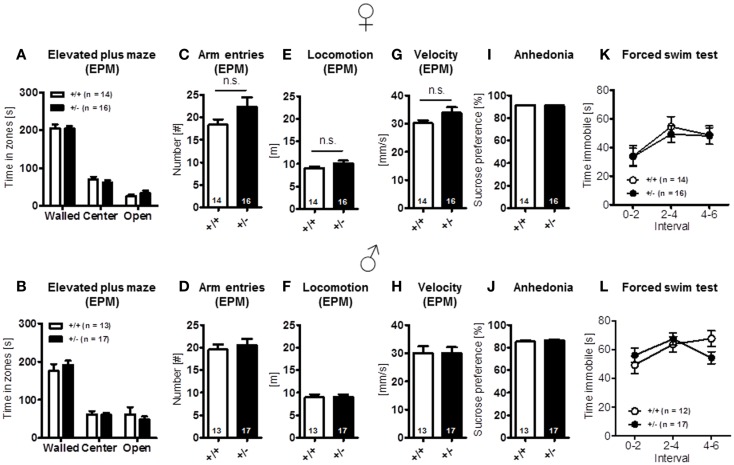
**Anxiety- and depression-relevant tests in *Ambra1*^+/−^ versus *Ambra1*^+^*^/^*^+^ mice show comparable results**. The upper row presents results for female, the lower row for male mice. **(A–H)** Elevated plus-maze readouts; **(I–L)** sucrose preference and forced swim test as readouts of depression. Mean ± SEM presented; respective sample sizes are indicated in the figures.

Finally, elevated plus-maze, a test of anxiety [females: time in zones: walled arms: *t*(28) = 0.079, *p* = 0.937, center: *t*(28) = 0.809, *p* = 0.425, open arms: *t*(28) = 1.022, *p* = 0.315, arm entries: *t*(28) = 1.635, *p* = 0.113, locomotion: *t*(28) = 1.627, *p* = 0.115, velocity: *t*(28) = 1.615, *p* = 0.117; males: time in zones: walled arms: *t*(28) = 0.779, *p* = 0.443, center: *t*(28) = 0.161, *p* = 0.873, open arms: *t*(28) = 0.748, *p* = 0.460, arm entries: *t*(28) = 0.512, *p* = 0.613, locomotion: *t*(28) = 0.082, *p* = 0.935, velocity: *t*(28) = 0.007, *p* = 0.994] (Figures 4A–H), and other determinants of emotionality, i.e., sucrose preference {anhedonia: [females: *t*(28) = 0.124, *p* = 0.902; males: *t*(28) = 1.267, *p* = 0.216]} (Figures 4I,J) and forced swim test (depression; Figures 4K,L), were essentially comparable in both genotypes [females: genotype: *F*(1, 28) = 0.078, *p* = 0.782, interval: *F*(2, 56) = 10.501, *p* < 0.001, genotype × interval: *F*(2, 56) = 0.200, *p* = 0.819; males: genotype: *F*(1, 27) = 0.034, *p* = 0.856, interval: *F*(2, 54) = 3.860, *p* = 0.027, genotype × interval: *F*(2, 54) = 3.860, *p* = 0.027]. In sum, these results suggest that *Ambra1* deficiency has no impairing effect on sensory functions, overall activity, and motor performance, the motivation to explore novel environments and emotionality in mice.

### Cognitive testing of *Ambra1*^+/−^ mice reveals impaired reversal learning in females only

**Figure 5:**
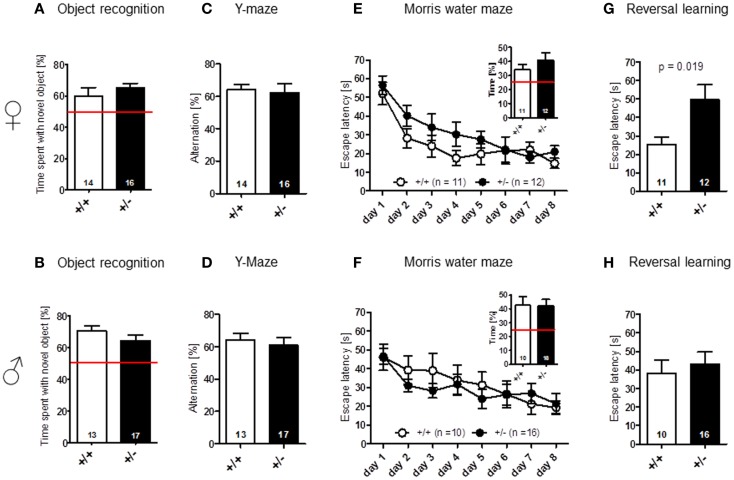
**Cognitive testing of male and female *Ambra1*^+/−^ versus *Ambra1*^+^*^/^*^+^ mice reveals impaired cognitive flexibility in female *Ambra1*^+/−^mice**. The upper row presents results for female, the lower row for male mice. **(A,B)** Novel object recognition, no-delay task. **(C,D)** Spatial alternation in Y-maze. **(E,F)** Spatial learning in the Morris water-maze; hidden platform acquisition curve presented. Figure inserts represent time spent searching for the platform in the target quadrant during the probe trial. **(G,H)** Reversal learning in the Morris water-maze. Mean ± SEM presented; respective sample sizes are indicated in the figures.

Novel object recognition (no-delay condition) [females: *t*(28) = 0.939, *p* = 0.356; males: *t*(28) = 1.180, *p* = 0.248] (Figures 5A,B), as well as spatial alternation in the Y-maze [females: *t*(28) = 0.255, *p* = 0.801; males: *t*(28) = 0.518, *p* = 0.608] (Figures 5C,D), were found to be comparable between genotypes. In Morris water-maze, hidden platform acquisition [females: genotype: *F*(1, 21) = 2.695, *p* = 0.116, days: *F*(7, 147) = 11.192, *p* < 0.001, genotype × days: *F*(7, 147) = 0.672, *p* = 0.696; males: genotype: *F*(1, 24) = 0.201, *p* = 0.658, days: *F*(7, 168) = 6.687, *p* < 0.001, genotype × days: *F*(7, 147) = 0.672, *p* = 0.696], as well as probe trial performance [females: *t*(21) = 1.065, *p* = 0.299; males: *t*(24) = 0.064, *p* = 0.949], were not significantly different between *Ambra1*^+/−^ mice and their WT littermates in both males and females (Figures 5E,F). However, female, but not male, *Ambra1*^+/−^ mice exhibited a significant impairment in the subsequently performed reversal learning test, that is thought to measure cognitive flexibility [overall reversal performance averaged over 4 days; females: *t*(21) = 2.553, *p* = 0.019; males: *t*(24) = 0.504, *p* = 0.6187] (Figures 5G,H).

### Autism-like phenotype detected in adult female *Ambra1*^+/−^ mice only

#### Communication – USV

**Figure 6:**
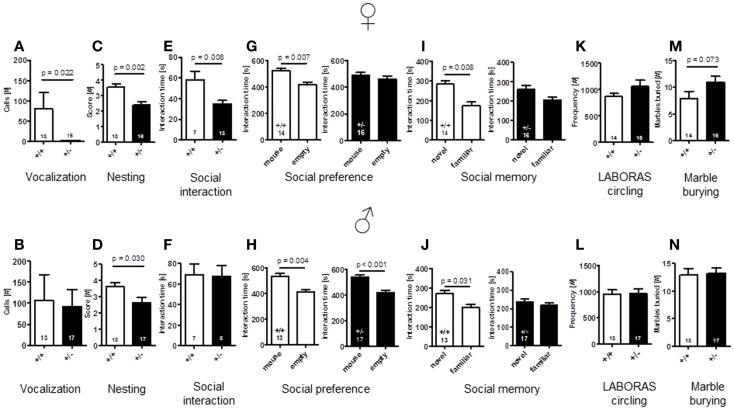
**Comparison of autism-readouts in *Ambra1*^+/−^ versus *Ambra1*^+^*^/^*^+^ mice of both genders discloses an autism-like phenotype in female *Ambra1*^+/−^mice**. The upper row presents results for female, the lower row for male mice. **(A,B)** Communication: ultrasound vocalization; **(C,D)** social competence: nesting score; **(E,F)** social interaction in pairs; **(G,H)** social preference tested in the tripartite chamber; **(I,J)** social memory tested in the tripartite chamber; **(K,L)** stereotypies/repetitive behaviors: circling, measured in LABORAS; and **(M,N)** stereotypies/repetitive compulsive behaviors: marble burying. Mean ± SEM presented; respective sample sizes are indicated in the figures.

To explore whether *Ambra1* deficiency would affect communication of male or female mice, we recorded the number of calls during a 3 min test. As compared to male mice with comparable vocalization of both genotypes [latency to call: *Ambra1*^+/−^: 102.94 ± 18.99 versus WT: 133.56 ± 20.44, *U*(28) = 92.000, *Z* = 0.856, *p* = 0.457, number of calls: *U*(28) = 91.000, *Z* = 0.902, *p* = 0.432], *Ambra1*^+/−^ females showed a longer latency to their first call [*Ambra1*^+/−^: 178.3 ± 1.7 versus WT: 99.2 ± 21.5, *U*(27) = 51.000, *Z* = 2.950, *p* = 0.020] and a significantly reduced number of calls [*U*(27) = 52.000, *Z* = 2.894, *p* = 0.022] (Figures 6A,B) as compared to their WT littermates. In fact, only 1 out of 16 *Ambra1*^+/−^ females vocalized at all, in contrast to 7 out of 13 WT females [Chi-square: 6.237, df = 1, *p* = 0.013]. We also investigated whether male *Ambra1*^+/−^ mice would display a more pronounced phenotype if exposed to an awake freely moving female instead of an anesthetized female. However, again no significant differences between male *Ambra1*^+/−^ and WT littermates were obtained (185.9 ± 56.3 versus 199 ± 62.6, *p* = 0.935; Mann–Whitney *U, n* = 15 per group). These results suggest that *Ambra1* deficiency impairs social communication only in female mice.

#### Nest building

An important domain of mouse social behavior, which has been found to be disturbed in autism-like phenotypes (Satoh et al., 2011; El-Kordi et al., 2013), is nest building. Regarding quality of nest building, i.e., nesting scores, both male and female *Ambra1*^+/−^ mice differed from their WT littermates [females: *t*(27) = 3.535, *p* = 0.002; males: *t*(28) = 2.288, *p* = 0.030] (Figures 6C,D). The same was true for the other nesting readout, weight of leftovers of nesting material [females: WT: 0.49 ± 0.14 versus *Ambra1*^+/−^: 1.29 ± 0.24, *U*(27) = 59.000, *Z* = 1.975, *p* = 0.050; males: WT: 0.41 ± 0.10 versus *Ambra1*^+/−^: 1.35 ± 0.29, *U*(28) = 62.000, *Z* = 2.033, *p* = 0.043].

#### Social approach/social interaction in pairs

In the social interaction test in pairs, unfamiliar mice of the same genotype were placed into a familiar testing cage and observed for 5 min. Whereas there was no significant difference in the time spent with social interactions between pairs of male *Ambra1*^+/−^ and WT mice [*t*(13) = 0.124, *p* = 0.903], the female *Ambra1*^+/−^ pairs showed significantly shorter interaction times as compared to the female WT pairs [*t*(18) = 2.955, *p* = 0.008] (Figures 6E,F).

#### Social interaction in the tripartite chamber: social preference and social memory

Whereas WT mice of both genders [females: *t*(13) = 3.233, *p* = 0.007, males: *t*(12) = 3.519, *p* = 0.004] and *Ambra1*^+/−^ males [*t*(16) = 4.165, *p* < 0.001] spent significantly more time in the compartment in which the stranger mouse was located as compared to the empty one, no such preference was observed in the female *Ambra1*^+/−^ mice [*t*(15) = 0.707, *p* = 0.490] (Figures 6G,H). As a measure of social memory, we investigated whether male and female *Ambra1*^+/−^ mice would discriminate between a familiar and an unfamiliar mouse. In this test, *Ambra1*^+/−^ mice of both genders failed to discriminate between familiar and unfamiliar mice [females: *t*(15) = 1.690, *p* = 0.112, males: *t*(16) = 0.643, *p* = 0.530], whereas WT mice of both genders preferred the unfamiliar over the familiar mouse [females: *t*(13) = 3.113, *p* = 0.008, males: *t*(12) = 2.439, *p* = 0.031] (Figures 6I,J). These results suggest that *Ambra1* deficiency in the mouse impairs social memory in both genders.

#### Circling and marble burying as equivalents of stereotypic and repetitive behaviors

As readout of stereotypic/repetitive behavior, frequency of circling in LABORAS was employed. Circling tended to be higher in female [*t*(28) = 1.350, *p* = 0.188], but not in male Ambra1^+/−^ mice [*t*(28) = 0.040, *p* = 0.968], as compared to their WT littermates (Figures 6K,L). In order to test whether *Ambra1* deficiency might have an influence on other readouts of stereotypic-like behavior, the marble burying test with an additional obsessive–compulsive component was performed. Once more, male mice did not show genotype differences [*t*(28) = 0.172, *p* = 0.864], while the number of marbles buried by female *Ambra1*^+/−^ mice tended to be higher as compared to their WT littermates [*t*(28) = 1.862, *p* = 0.073] (Figures 6M,N). These results suggest that *Ambra1* deficiency slightly increases stereotypic behavior exclusively in female *Ambra1*^+/−^ mice.

#### Autism severity composite score

**Figure 7:**
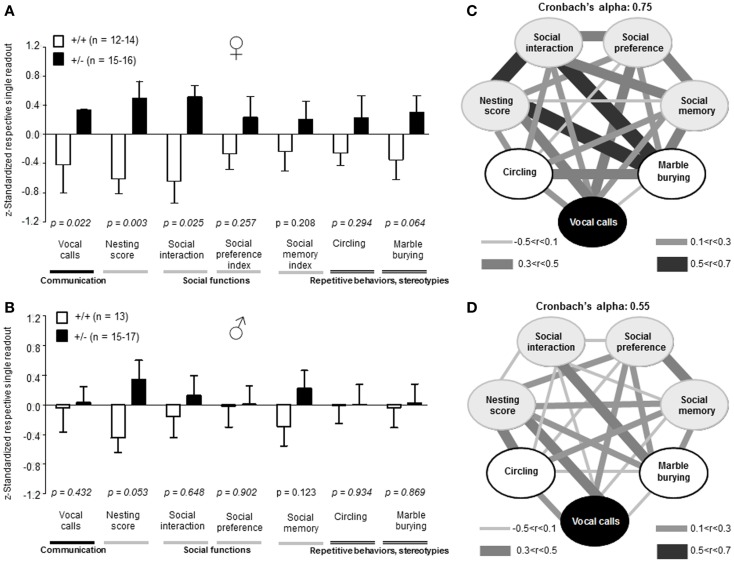
**Composition and intercorrelations of the autism severity score in *Ambra1*^+/−^ versus *Ambra1*^+^*^/^*^+^ mice of both genders are presented**. The upper row presents results for female, the lower row for male mice. **(A,B)** Presentation of *z*-standardized autism-relevant readouts to be integrated into the composite score; mean ± SEM presented; respective sample sizes are indicated in the figures. **(C,D)** Intercorrelation pattern of the autism-relevant readouts including Cronbach’s alpha as a quality measure for internal consistency of the autism severity composite score.

**Figure 8:**
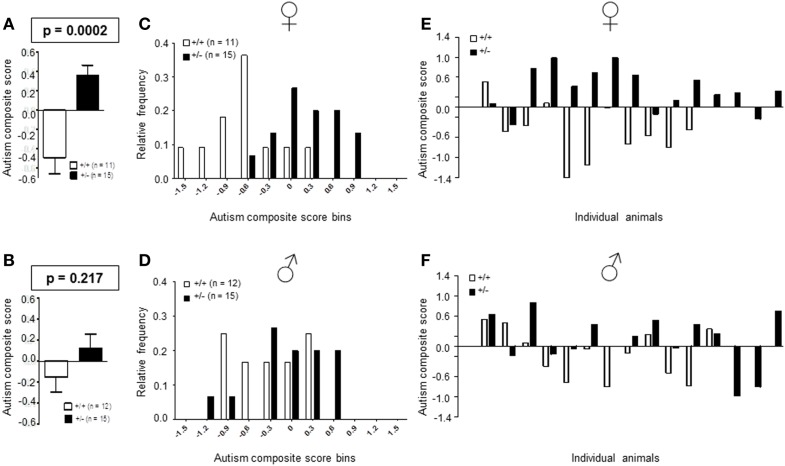
**Autism composite score results for *Ambra1*^+/−^ versus *Ambra1*^+^*^/^*^+^ mice of both genders underline the female autism-like phenotype in *Ambra1*^+/−^**. The upper row presents results for female, the lower row for male mice. **(A,B)** Highly significant genotype-dependent score difference in female but not male mice; mean ± SEM presented. **(C,D)** Frequency distribution of autism composite score bins dependent on genotype; **(E,F)** Composite score presentation of all individual animals reveals a clear discrimination between genotypes in female but not male mice.

Recently, we developed an autism severity composite score that accounts for individuality of discrete symptom severity in the autistic syndrome as a whole (El-Kordi et al., 2013). For this autism severity composite score, selected single readouts, covering all three main diagnostic domains (communication, social interaction, and stereotypies/repetitive behaviors) are *z*-standardized and genotype groups contrasted by Mann–Whitney *U*-tests (Figures 7A,B; Tables 1 and 2; Box 1). The composite score reflects the overall severity of autistic behaviors in a continuous fashion with higher values indicating higher severity of autistic behaviors. For both male and female mice, the optimal composite score was based on vocal calls, nesting, social interactions in pairs, social preference, social memory, circling, and marble burying. A reliability analysis of the readouts selected for the composite score yielded Cronbach’s α = 0.75 for female and α = 0.55 for male mice. The corresponding intercorrelations between single readouts are displayed in Figures 7C,D. The between-group comparison of the autism severity composite score, calculated by integrating the means of all behavioral readouts, was significantly different between female [*U*(24) = 16.000, *Z* = 3.452, *p* = 0.0002] but not male [*U*(25) = 64.000, *Z* = 1.245, *p* = 0.217] mice (Figures 8A,B). As further depicted in Figures 8C,D, the distribution of the relative frequency with which a certain score range is evident in either the *Ambra1*^+/−^ or *Ambra1*^+^*^/^*^+^ group, discriminates very well between genotypes in females. There is only a slight overlap in the frequency distribution in female groups for the intermediate score range, with *Ambra1*^+/−^mice showing a right-ward shift toward higher scores and *Ambra1*^+^*^/^*^+^ mice showing a left-ward shift toward lower scores. In contrast, the male groups display a much greater overlap with only a weak right-ward shift evident in male *Ambra1*^+/−^subjects (Figures 8C,D). These findings are also nicely illustrated by the composite scores achieved by individual mice (Figures 8E,F).

**Table 1:** **Individual *z*-standardized values for single readouts of the autism composite score of female *Ambra1*^+/+^ and *Ambra1*^+/−^*mice* (see Figure 7)**.

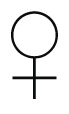 , +/+ Mouse	Vocal calls	Nesting score	Social interaction	Social preference	Social memory	Circling	Marble burying
1	0.34173	− 0.09881	0.75491	− 0.07682	1.99546	0.01319	0.73121
2	0.34173	− 1.05399	− 0.18438	0.4353	−0.03264	− 0.7934	− 1.82447
3	0.34173	− 0.09881		0.69136	0.10723	0.45134	− 0.97258
4	− 2.37401	− 0.09881	0.75491	0.4353	−0.52218	0.16007	− 0.7596
5	0.32267	− 0.09881	− 0.18438	0.94742	1.08632	− 1.22408	− 0.12068
6	− 4.26074	− 1.05399	− 1.49938	− 1.61317	−0.80192	− 0.99754	0.30526
7	− 1.14478	− 2.00916	− 1.49938	− 0.71697	−0.52218	− 0.87058	− 1.18555
8	0.34173	0.85636		− 1.22908	−1.57119	0.35177	− 0.33366
9	− 0.40153	− 1.05399	0.00348	0.17924	0.31704	0.21733	0.73121
10	0.34173	− 1.05399	− 1.82814	− 0.46091	−0.80192	0.50611	− 1.61149
11	0.09397	− 1.05399	− 1.45242	− 0.20485	−1.01172	0.18497	− 0.33366
12	0.34173	− 1.05399	− 1.82814	− 1.48514	0.73664	− 1.15687	− 0.97258
13	0.28455	− 0.09881	− 1.45242	− 0.07682	−1.01172	0.01319	− 0.54663
14			0.70795	− 0.58894	−1.29146	− 0.49217	2.00904
*Mean*	−*0.417653*	−*0.61314*	−*0.642283*	−*0.26886*	−*0.237446*	−*0.25976*	−*0.34887*
±*SEM*	*0.375968*	*0.197575*	*0.290571*	*0.204794*	*0.25797*	*0.163004*	*0.265641*

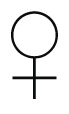 , **+/−Mouse**	**Vocal calls**	**Nesting score**	**Social interaction**	**Social preference**	**Social memory**	**Circling**	**Marble burying**

1	0.34173	− 0.09881	1.31848	− 1.61317	−0.66205	0.8646	0.51823
2	0.34173	0.85636	1.22455	− 0.46091	−0.94178	− 2.1676	− 0.97258
3	0.34173	0.85636	1.31848	2.09969	0.87651	− 0.36521	0.51823
4	0.34173	− 0.09881	1.22455	1.20348	1.22618	1.50689	1.58310
5	0.34173	0.85636	0.61402	− 1.10105	0.0373	2.08943	0.30526
6	0.34173	− 0.09881	0.56705	1.58757	2.4850	− 0.25816	0.30526
7	0.34173	1.81154	0.61402	0.81939	−0.03264	1.73841	1.79607
8	0.34173	1.81154	0.56705	− 0.20485	−0.03264	1.51187	0.73121
9	0.30361	− 0.09881	− 0.04349	0.17924	−0.87185	0.08539	− 0.33366
10	0.34173	0.85636	− 0.04349	0.30727	−0.24244	0.25716	− 0.33366
11	0.34173	− 0.09881	0.61402	0.56333	1.36605	1.08865	0.09229
12	0.34173	1.81154	0.61402	− 0.20485	−0.80192	− 0.96518	1.15715
13	0.34173	− 1.05399	− 0.27831	2.09969	0.17717	− 0.11377	0.94418
14	0.34173	− 1.05399	− 0.32527	0.17924	0.0373	0.13767	− 0.7596
15	0.34173	0.85636	− 0.27831	− 0.46091	1.29612	− 0.03162	0.73121
16	0.34173	0.85636		− 1.22908	−0.59211	− 1.7419	− 1.39852
*Mean*	*0.339348*	*0.498172*	*0.513825*	*0.235255*	*0.207763*	*0.227289*	*0.305261*
±*SEM*	*0.002307*	*0.221368*	*0.148043*	*0.271585*	*0.23780*	*0.293291*	*0.216275*

**Table 2:** **Individual *z*-standardized values for single readouts of the autism composite score of male *Ambra1*^+/+^ and *Ambra1*^+/−^*mice(see Figure 7)***.

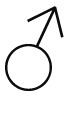 , +/+ Mouse	Vocal calls	Nesting score	Social interaction	Social preference	Social memory	Circling	Marble burying
1	0.51868	0.05421	0.95404	− 0.2625	0.91895	0.60432	1.00313
2	0.51868	− 0.7589		− 1.0660	−1.6322	0.13044	0.23803
3	0.51868	0.05421	0.95404	1.02312	−0.3978	− 0.1111	1.25816
4	0.51338	0.05421	0.31274	1.50522	−0.4801	− 1.0160	− 0.2720
5	0.51868	0.05421	− 1.5399	0.38032	−0.3155	− 0.4535	− 1.2922
6	− 2.5107	− 0.7589	0.31274	− 1.2267	0.75436	− 1.1444	− 0.0170
7	0.51868	0.05421	− 1.5399	1.02312	1.08354	− 0.9029	− 0.5271
8	− 2.6804	− 1.5720	− 0.9699	0.54102	−0.4801	− 0.5269	0.49306
9	0.51868	− 0.7589	− 0.5780	0.21962	−1.3030	− 0.0102	1.00313
10	0.51868	0.86728	− 0.9699	− 0.1018	−0.3978	1.83333	− 0.0170
11	0.51868	− 0.7589	− 0.5780	− 2.0302	−0.7269	− 0.2303	0.23803
12	− 0.5477	− 1.5720	0.84716	− 0.7446	−1.7145	1.02316	− 2.3123
13	0.51868	− 0.7589	0.88279	0.54102	0.91895	0.69298	− 0.2720
*Mean*	−*0.0429*	−*0.4461*	−*0.1593*	−*0.0152*	−*0.2901*	−*0.0085*	−*0.0366*
±*SEM*	*0.31198*	*0.18843*	*0.25944*	*0.2723*	*0.25564*	*0.23045*	*0.25946*

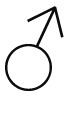 , **+/−Mouse**	**Vocal calls**	**Nesting score**	**Social interaction**	**Social preference**	**Social memory**	**Circling**	**Marble burying**

1	0.51868	0.86728	1.80912	− 0.5839	0.1783	0.65935	1.00313
2	− 1.4231	− 0.7589	0.20585	0.54102	0.09601	0.20076	0.23803
3	0.51868	0.86728	1.80912	1.02312	1.41271	1.09042	− 0.5271
4	0.51868	0.05421	0.20585	0.21962	−0.2332	− 1.7895	− 0.0170
5	0.42849	1.68036	− 1.1837	− 0.7446	0.67207	0.48203	− 1.5472
6	0.51868	− 0.7589	− 1.1837	2.46942	1.90647	0.19159	− 0.0170
7	0.51868	0.86728	0.81153	0.05892	−1.7968	− 0.585	0.23803
8	0.51868	− 0.7589	0.95404	0.38032	−0.3978	0.35974	0.49306
9	0.51868	1.68036	− 0.2929	0.70172	0.91895	− 0.5941	0.74810
10	0.20036	− 0.7589	0.81153	0.38032	−0.2332	− 1.6122	1.00313
11	− 0.4416	0.86728	0.95404	− 0.9053	0.34289	0.5340	1.76823
12	− 0.6485	0.05421	− 0.2929	0.21962	1.74189	0.93144	− 0.0170
13	0.38605	− 0.7589	− 0.8274	− 1.7088	−1.5499	− 0.9793	− 1.2922
14	0.06243	− 1.5720	− 1.0411	− 1.2267	0.01372	− 0.3801	− 1.2922
15	0.51868	1.68036	− 0.8274	0.70172	0.34289	2.61905	− 0.0170
16	− 2.6645	0.86728		− 0.1018	0.75436	0.4484	− 1.8022
17	0.50807	1.68036		− 1.2267	−0.3978	− 1.4654	1.5132
*Mean*	*0.03278*	*0.34117*	*0.12747*	*0.01166*	*0.22187*	*0.00653*	*0.0280*
±*SEM*	*0.20805*	*0.25255*	*0.24214*	*0.23868*	*0.23548*	*0.26322*	*0.24672*

### Aspects of an autism-like phenotype are already seen in female Ambra1^+/−^ pups

**Figure 9:**
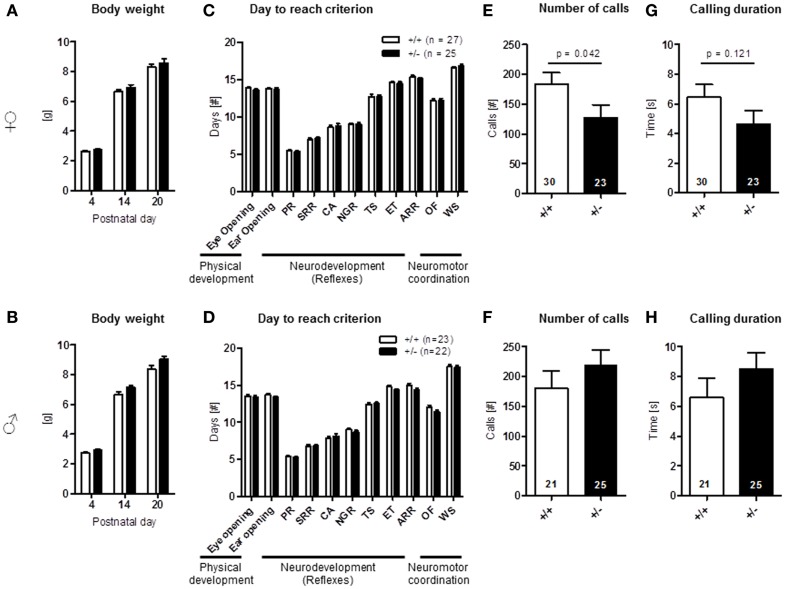
**Neonatal development and pup vocalization in *Ambra1*^+/−^ versus *Ambra1*^+^*^/^*^+^ mice uncover early ultrasound communication deficits in females**. The upper row presents results for female, the lower row for male mice. **(A,B)** Body weight over time is comparable between genotypes; **(C,D)** no genotype differences in physical development, neurodevelopment (PR, placing response; SRR, surface righting reflex; CA, cliff avoidance; NGR, negative geotaxis reflex; TS, tactile startle; ET, ear twitch; ARR, air righting reflex), and neuromotor coordination (OF, open field traversal; WS, wire suspension). Overall test results are expressed as days to reach criterion. **(E–H)** Ultrasound vocalization of pups briefly separated from their mother on postnatal day 8/9. Mean ± SEM presented; respective sample sizes are indicated in the figures.

Physical development, including body weight [females: genotype: *F*(1, 50) = 0.900, *p* = 0.347, PND: *F*(2, 100) = 1464.098, *p* < 0.001, genotype × PND: *F*(2, 100) = 0.390, *p* = 0.678; males: genotype: *F*(1, 43) = 3.935, *p* = 0.054, PND: *F*(2, 86) = 1540.385, *p* < 0.001, genotype × PND: *F*(2, 86) = 2.331, *p* = 0.103], eye opening [females: *U*(60) = 391.000, *Z* = 1.318, *p* = 0.187, males: *U*(45) = 208.000, *Z* = 1.162, *p* = 0.245], ear opening [females: *U*(60) = 400.500, *Z* = 1.154, *p* = 0.248, males: *U*(45) = 203.000, *Z* = 1.236, *p* = 0.217], the development of neurological reflexes [females: genotype: *F*(1, 60) = 0.084, *p* = 0.772, PND: *F*(6, 360) = 627.766, *p* < 0.001, genotype × PND: *F*(6, 360) = 0.294, *p* = 0.940; males: genotype: *F*(1, 43) = 2.028, *p* = 0.162, PND: *F*(6, 258) = 689.532, *p* < 0.001, genotype × PND: *F*(6, 258) = 1.388, *p* = 0.220], and the development of neuromotor coordination [open field traversal, females: *U*(60) = 465.500, *Z* = 0.157, *p* = 0.875, males: *U*(45) = 218.500, *Z* = 0.957, *p* = 0.339], wire suspension [females: *U*(60) = 408.500, *Z* = 0.994, *p* = 0.320, males: *U*(45) = 245.000, *Z* = 0.188, *p* = 0.851] were comparable in pups of both genders and genotypes (Figures 9A–D). In contrast, ultrasound vocalization upon short separation from mothers on PND 8/9 revealed a reduction in number of calls [*U*(51) = 231.000, *Z* = 2.037, *p* = 0.042] and a trend for a reduced duration of calls [*U*(51) = 237.500, *Z* = 1.550, *p* = 0.121] in female *Ambra1*^+/−^ mice compared to their WT littermates. In the males, no such genotype differences were observed [number: *U*(44) = 215.500, *Z* = 1.037, *p* = 0.300; duration: *U*(44) = 210.000, *Z* = 1.158, *p* = 0.247] (Figures 9E–H).

### Stronger reduction from WT levels of Ambra1 protein in the cortex of *Ambra1*^+/−^ females compared to males

**Figure 10:**
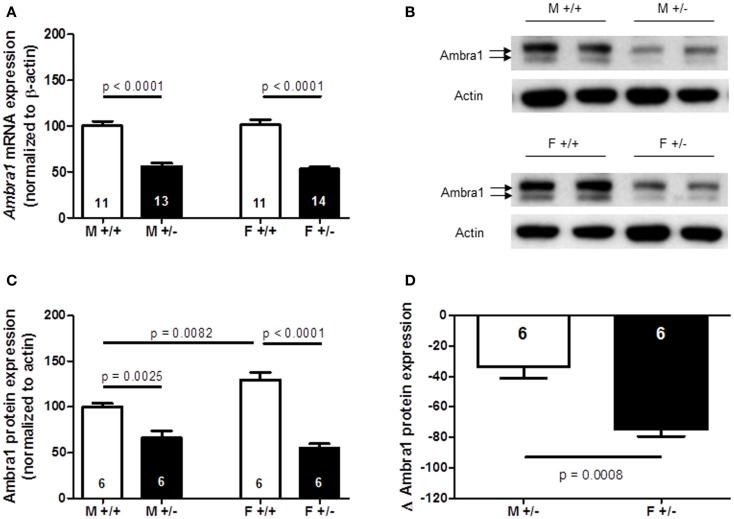
**Quantification of *Ambra1* mRNA and protein in cortex of male and female *Ambra1*^+/−^ versus *Ambra1*^+^*^/^*^+^ mice delivers a first hint to potentially explain the behavioral gender difference**. **(A)** mRNA; **(B)** sample western blot; **(C)** western blot quantification; **(D)** note the significantly higher relative reduction (delta) from the respective wildtype control (*Ambra1*^+^*^/^*^+^) of Ambra1 protein in cortex of female as compared to male *Ambra1*^+/−^ mice. M, male; F, female; Mean ± SEM presented; respective sample sizes are indicated in the figures.

Together, the results of autism-like phenotype testing are in line with the observation of a marked female autistic phenotype in *Ambra1*^+/−^ mice, and only mild subthreshold autistic symptoms (nesting score, social memory) in male *Ambra1*^+/−^ mice. In a first approach to understand this sexual dimorphism, *Ambra1* mRNA and protein levels were quantified in the cortex of male and female mice. As shown in Figures 10A–C, both mRNA [females: *t*(23) = 9.165, *p* < 0.0001; males: *t*(22) = 7.068, *p* < 0.0001] and protein levels [females: *t*(10) = 7.993, *p* < 0.0001; males: *t*(10) = 3.996, *p* = 0.0025] were distinctly reduced in *Ambra1*^+/−^ mice of both genders as compared to WT mice. Interestingly, Ambra1 protein (but not mRNA) expression in female WT mice was higher as compared to males [*t*(10) = 3.288, *p* = 0.0082], and the relative reduction in females from their respective WT control level was much more pronounced than in males [females: *t*(10) = 4.743, *p* = 0.0008] (Figure 10D). This difference may give a first explanation on why females but not males display the autism-like phenotype.

## Discussion

In the present study, we show an autism-like phenotype in a mouse line, deficient of a central protein, Ambra1, in the autophagy cascade. Importantly, we find that this phenotype is essentially restricted to female *Ambra1*^+/−^ mice, both adults and neonates, and only marginally present in *Ambra1*^+/−^ males (nesting score, social memory). Moreover, we identify features of reduced cognitive flexibility exclusively in female *Ambra1*^+/−^ mice.

The observed multifaceted autism-like syndrome in females consists of distinctive symptoms for all three lead features of the disorder: disturbed social interactions, compromised verbal (ultrasound) communication, and repetitive behaviors/restricted interests. Based on these typical behavioral symptoms, we calculated for *Ambra1*^+/−^ mice an autism severity composite score, as it had originally been developed for autism severity estimation in *Nlgn4*^−/−^ mice (El-Kordi et al., 2013). This score allowed again a highly significant discrimination between autistic and non-autistic mice.

Since autism is usually diagnosed in children before the age of 3 years, assessment of neurobehavioral developmental milestones and early ultrasound communication in neonatal mice up to the age of 3–4 weeks has been important for further characterization of the *Ambra1*^+/−^ mouse model of autism. Indeed, an autistic phenotype as revealed by reduced vocalizations was uncovered in pups and again found restricted to the female gender.

As a first clue to why females might be more severely affected by autism-like features, we detected a more pronounced reduction in Ambra1 protein from WT level in females as compared to males. In fact, basic Ambra1 expression in female WT mice is higher as compared to males, potentially indicating a greater requirement of this protein in females for fully functional autophagy. Along these lines, a recent paper reported that neonatal female rats have higher basal autophagy activity in the brain as compared to males (Weis et al., 2014). This hint of a sexual dimorphism regarding autophagy, and thus basal turnover of cellular components including synapses, is definitely worth pursuing. It may even help to explain certain basic male/female differences regarding synapse function and synaptopathies. However, at this point, it cannot entirely be excluded that as yet unknown *Ambra1* functions exist to explain the gender difference that are unrelated to autophagy.

Of note, the overall male/female ratio in autism is about 4:1. So far, to our knowledge, no genotype has been reported to cause an autism-like phenotype exclusively in females even though gender-specific findings have been described in several other mouse models of autism (e.g., Laarakker et al., 2012; Schmeisser et al., 2012; Tilot et al., 2014). For example, it has been reported that a knock-in of a cytoplasm-predominant Pten protein (*Ptenm^3m4/m3m4^* mice) induces increases in social preference and memory selectively in homozygous male but not female *Ptenm^3m4/m3m4^* mice. These gender-specific differences in social behavior were associated with impaired motor coordination but normal recognition memory (Tilot et al., 2014). However, although a number of morphological, cellular and biochemical changes were detected in the brains of the *Ptenm^3m4/m3m4^* mice, none of these changes turned out to be gender-dependent. Although the gender differences in ASD may have a genetic basis, the identification of mechanisms that lead to gender-specific symptoms remains a challenging task for the future.

In humans, corresponding to mice, autistic symptom severity and distribution show gender differences (Hartley and Sikora, 2009; Baron-Cohen et al., 2011; Bolte et al., 2011; Rinehart et al., 2011; Beacher et al., 2012; Zwaigenbaum et al., 2012). But not only gender, also individuality is an important issue in autism. ASD are multifaceted conditions, composed of severe socio-communicative deficits, restricted interests, stereotypies, and repetitive behaviors. Like humans, also individual mice vary considerably in the relative severity of the respective symptom categories. When looking at the standard deviations in autism tests obtained with our test battery, there is clear interindividual variation in the expression of distinct symptoms. The application of the autism severity score may therefore deliver a more robust ground for future experimental treatment studies than single readouts. The score integrates all relevant symptoms in the evaluation of individual syndrome severity. This way, it also provides a measure for future comparison of “autistic mouse lines” with each other. Such comparison will go hand in hand with an extended validation of the score in other established mouse models of autism.

In the mouse model studied here, a single gene aberration, *Ambra1*^+/−^, causes an autism-like syndrome in females. Therefore, ideally, a causal treatment should also combat all features of the disorder. First experimental therapeutic approaches may involve a targeted modulation of autophagy, assuming that the phenotypical consequence of a deficiency in *Ambra1* is predominantly related to its involvement in autophagy. Even then, it will remain to be determined if autophagy-related mechanisms can be efficiently influenced by non-toxic pharmacological tools such that a permanent rescue from the autism-like symptoms in female *Ambra1*^+/−^ mice will ensue and open ways for later application in human ASD.

On the other hand, the subthreshold phenotype found in males may stimulate experimental work trying to illuminate modifying environmental factors that might lead to the development of an overt autistic syndrome in *Ambra1*^+/−^ males as well. Such knowledge on environmental and thus perhaps avoidable risk could facilitate the initiation of preventive measures in endangered individuals.

## Author Contribution

Derek Lu, Kurt Hammerschmidt, Anes Ju, and Martesa Tantra performed all behavioral analyses on adult and neonatal mice. Liane Dahm, together with Anes Ju, conducted the biochemical analyses. Ekrem Dere, Liane Dahm, Anne Kästner, Kurt Hammerschmidt, Martesa Tantra, and Hannelore Ehrenreich performed the statistical analyses, designed the figures and interpreted the final data. Hannelore Ehrenreich, supported by Kamal Chowdhury, planned, supervised, and coordinated the project. Hannelore Ehrenreich and Ekrem Dere wrote the manuscript. All authors contributed to the current version of the paper.

## Conflict of Interest Statement

The authors declare that the research was conducted in the absence of any commercial or financial relationships that could be construed as a potential conflict of interest.
